# Pedigree analysis and genetic inheritance of fatal familial insomnia (FFI) in a Portuguese multigenerational family

**DOI:** 10.1007/s00415-025-13432-2

**Published:** 2025-10-17

**Authors:** Angela Da Silva Correia, Ines Laginha, Susana Guimaraes, Kathrin Dittmar, Matthias Schmitz, Joaquin Castilla, Inga Zerr, Susana Da Silva Correia

**Affiliations:** 1 Department of Neurology, University Medical Center and the German Center for Neurodegenerative Diseases (DZNE), Georg-August University, Goettingen, Germany; 2 APDP-Associação Portuguesa das Doenças Priónicas, Santa Maria da Feira, Portugal; 3 High-Risk Autopsy Unit, Biosafety Level 3(BSL-3), Department of Pathology, ULSSJoão, Porto, Portugal; 4 Faculty of Medicine, University of Porto, Porto, Portugal; 5 Centro de Investigación Cooperativa en Biociencias (CIC BioGUNE), Basque Research and Technology Alliance (BRTA), 48160 Derio, Bizkaia Spain; 6 Centro de Investigación Biomédica en Red de Enfermedades Infecciosas (CIBERINFEC), Carlos III National Health Institute, 28029 Madrid, Spain; 7 Basque Foundation for Science, IKERBASQUE, 48009 Bilbao, Bizkaia Spain

**Keywords:** Fatal familial insomnia, FFI, Prion diseases, Portugal, PrPC, PrPSc

## Abstract

Fatal familial insomnia (FFI) is a rare, autosomal dominant prion disease caused by a mutation in the PRNP gene, leading to the misfolding of the cellular prion protein (PrPC) into its pathogenic form (PrPSc). This results in neurodegeneration, particularly in the thalamus, a key region regulating sleep–wake cycles, which underlies the hallmark symptoms of FFI, including insomnia, autonomic dysfunctions, motor disturbances and cognitive decline. This study focuses on a Portuguese family with FFI, providing a detailed pedigree analysis spanning five generations and comprising 134 individuals, to elucidate inheritance patterns, disease onset, and clinical progression. The findings confirm the autosomal-dominant inheritance pattern and a strong familial clustering of the disease with age of onset in the late 50s (mean 57 years). Although 67% of affected individuals succumbing to the disease within months to 1.5 years, a notably 33% exhibited prolonged survival beyond the typical disease duration, exceeding proportions reported in the literature. Family members retrospectively reported prodromal symptoms, including generalized pain, headaches, tinnitus, pruritus, and behavioral changes, occurring up to five years before diagnosis. In several cases, reportedly, disease onset was associated with major phycological stressors (e.g., emotional stress or mourning). While the significance of these observations remains uncertain, they may provide insights into potential early features in this kindred. Further research integrating genomic sequencing, biomarkers, and longitudinal clinical assessments are needed to better understand the mechanisms underlying the heterogeneity of FFI and to explore potential therapeutic interventions.

**Supplementary Information:**

The online version contains supplementary material available at 10.1007/s00415-025-13432-2.

## Introduction

Fatal familial insomnia (FFI) is a rare genetic prion disease clinically characterized by severe, progressive untreatable insomnia, autonomic dysfunction, motor disturbances, and rapid cognitive impairment, ultimately leading to death within months to three years after disease onset [1].

FFI is caused by an autosomal dominant mutation in the PRNP gene, which encodes the cellular prion protein (PrPC). This mutation results in the substitution of aspartic acid for asparagine at the position 178 (D178N) within the PrPC [2]. PrPC is a glycoprotein predominantly expressed in the central nervous system, particularly in neurons [3]. While the exact physiological role of PrPC is not fully understood, it has been implicated in synaptic transmission, neuroprotection, and regulation of oxidative stress [4, 5].

In individuals carrying the FFI mutation, the structural stability and cellular processing of PrPC are altered, alterations that are associated with the pathogenic processes underlying the diseases [6, 7]. The pathogenic isoform PrPSc exhibits resistance to protease degradation and has the ability of self-replication by inducing the conversion of native PrPC into this aberrant conformation (PrPSc) [8]. This misfolded protein accumulates and aggregates within the central nervous system, disrupting cellular homeostasis and triggering neurodegeneration [9]. The Thalamus, a critical brain region essential for regulating sleep–wake cycles and sensory processing, is one of the earliest affected brain regions leading to the hallmark symptoms of FFI [1, 10]. First described in Italian families, FFI has since been identified in multiple populations worldwide, including Italian, Spanish, Chinese, Japanese, United States, Moroccan, and Australian pedigrees, further reinforcing its highly penetrant inheritance pattern [1, 11–18]. To date, over 70 families worldwide have been reported to be affected by this rare disease, with individuals showing variable geographical distribution and frequency across different regions [19, 20]. The majority of the affected families have European ancestry, with cases reported in countries such as Spain, Austria, Germany, Italy, UK, France, Finland [13, 21–27]. With most of the cases more prevalent in certain regions such in Spain and Germany, where it accounts 56.8% and 25% of genetic prion diseases are FFI. In Asia, particularly in China, where cases have been increasing in recent years, FFI represents 43.5% of all genetic prion diseases [28].

In this study, we conduct a pedigree analysis of a Portuguese family affected by FFI to determinate the age onset and disease duration among affected individuals, aiming to evaluate the potential impact of the age onset in disease progression, gender, and identify prodromal symptoms that may precede the onset of the disease. In addition, by comparing these findings with the previous documented FFI pedigrees, the study aims to elucidate potential disease progression and phenotypic variations across different genetic backgrounds.

## Materials and methods

### Pedigree construction

A genealogical study was conducted on a Portuguese family affected by FFI. The pedigree data were collected retrospectively from interviews with family members, historical records, and medical documentation. The family tree spans five generations, with 125 individuals represented. Owing to privacy concerns, the data from the two most recent generations (6 and 7) were excluded. The pedigree includes 82 individuals belonging to the same family, spouses who married into the family were documented but not included in the core analysis. The tree is considered as complete as possible, but some individuals from the first and second generations may be missing due to historical data limitations. The family tree was built using pedsuite packages in R computing.

### Data collection

Age of death, disease duration, age of onset, and clinical symptoms were collected. The age of onset was estimated by subtracting the disease duration from the age of death. The data were obtained through family interviews, and clinical records when available. All reported symptoms and observed clinical signs were based on information provided by family members and caregivers of the patients.

### Statistical analysis

The distribution of age at onset and disease duration was analyzed using descriptive statistics. The data were analyzed using Python 3 for statistical computing.

For the analysis of the correlation between age at onset and disease duration, disease duration was converted from months to years for analysis. Cases with missing age of onset data were excluded, resulting in 27 cases for correlation analysis. Normality of variables was assessed using the Shapiro–Wilk test. The correlation between age of onset and disease duration was analyzed using Spearman’s rank correlation, as disease duration was not normally distributed (*p* = 0.0345). Statistical significance was set at *p* < 0.05. Analyses were performed using Python 3.

The age at death across generations was first assessed for normality using the Shapiro–Wilk test. Because Generation IV data were not normally distributed (*p* = 0.0261), we applied a nonparametric approach, Kruskal–Wallis with Dunn’s multiple comparisons test.

## Results

### Family pedigree of the Portuguese family

**Fig. 1:**
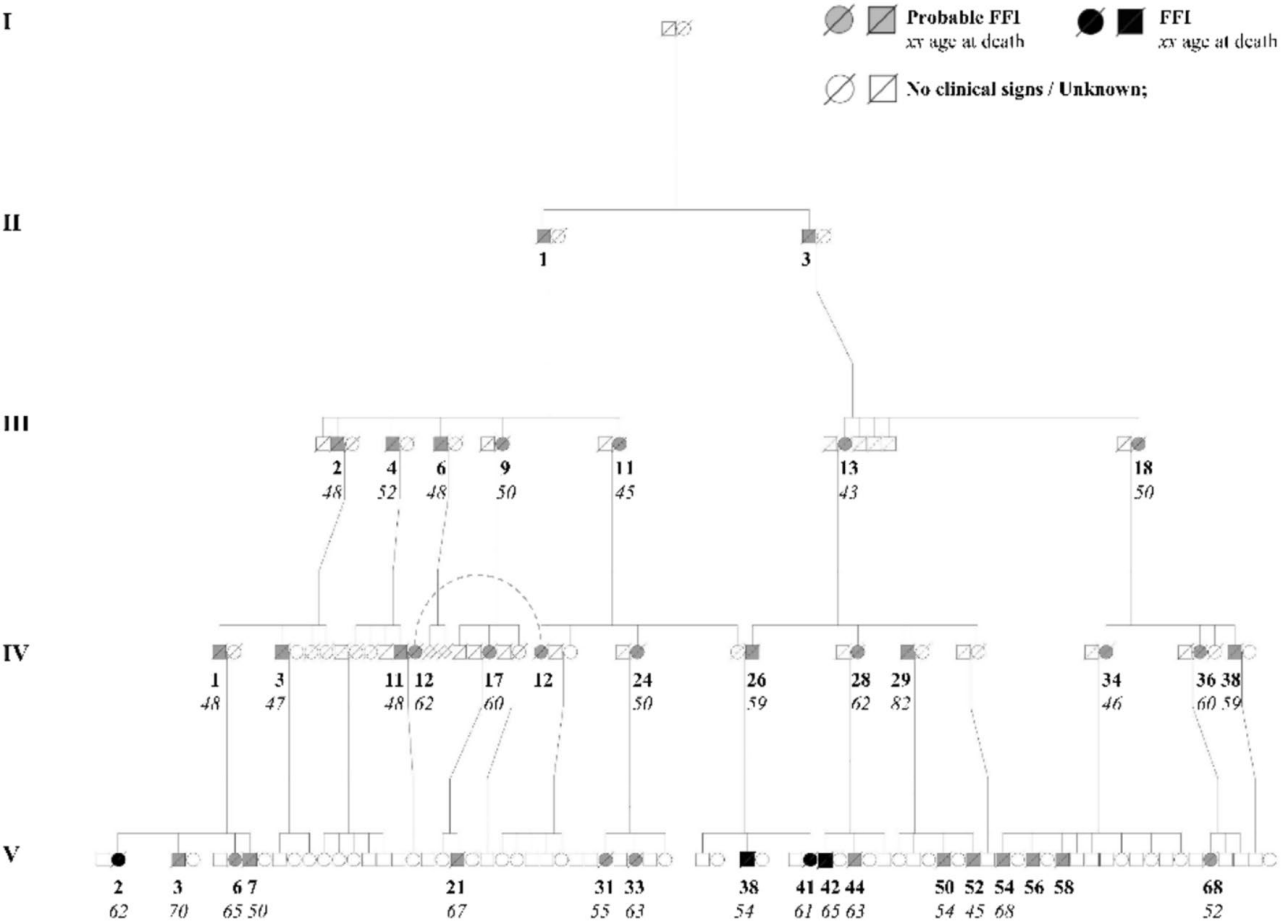
Pedigree of a Portuguese family with FFI. Squares represent males (*n* = 70), and circles represent females (*n* = 64). Probable and definite FFI individuals are indicated by gray-filled (probable) and black-filled (definite) symbols (*n* = 38), while unaffected individuals are unfilled. Deceased individuals are marked with a diagonal line. The approximate year of birth of the oldest individual in generation II is ~ 1880, and the youngest in generation V is ~ 1975. Birth years show wide variability within and across generations, with considerable overlap

The family pedigree (Fig. 1) presents a comprehensive analysis of the inheritance pattern of FFI within a Portuguese family, comprising 134 individuals, with a total of 70 males (represented by squares), 64 females (represented by circles), and 2 individuals of unknown sex (represented by rhombuses). The autosomal dominant inheritance of FFI is demonstrated by the presence of affected individuals across all generations. In total, 38 individuals have been identified as affected by the condition, consisting of 22 males (denoted by black squares) and 16 females (denoted by black circles), all of whom are deceased.

**Fig. 2:**
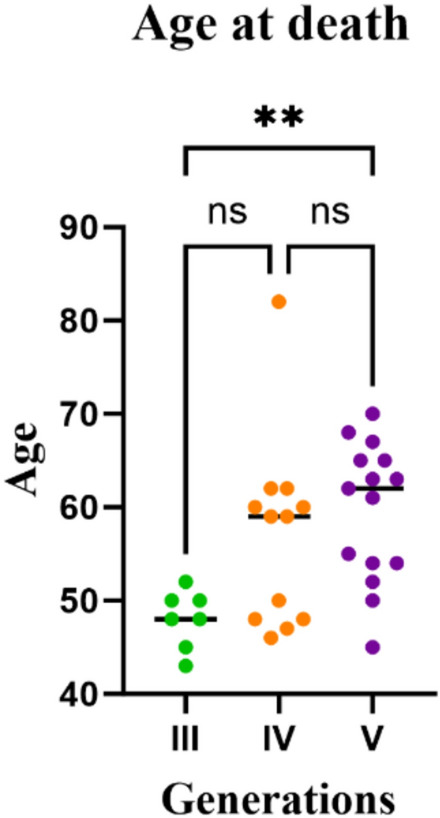
Age at death across three generations of a Portuguese pedigree family. Age at death for individual in generation III (*n* = 7) is represented by green circles, generation IV (*n* = 12) by orange circles, and generation V (*n* = 15) by violet cycles. Non-parametric comparisons were performed (Kruskal–Wallis with Dunn’s multiple comparisons test) given non-normal distribution in Generation IV (Shapiro–Wilk test, *p* = 0.0261). A significant difference in age at death was observed between generation III and V (**p = 0.0043)

A significant increase in age at death was observed between generations 3 and 5 (**p = 0.0043) (Fig. 2). However, it is important to note that additional affected individuals may exist, but have not yet been identified, and it remains possible that more cases could emerge within the family. Owing to privacy concerns, data from the two most recent generations (6 and 7) were excluded.

### Age of onset and disease duration in affected individuals

**Fig. 3:**
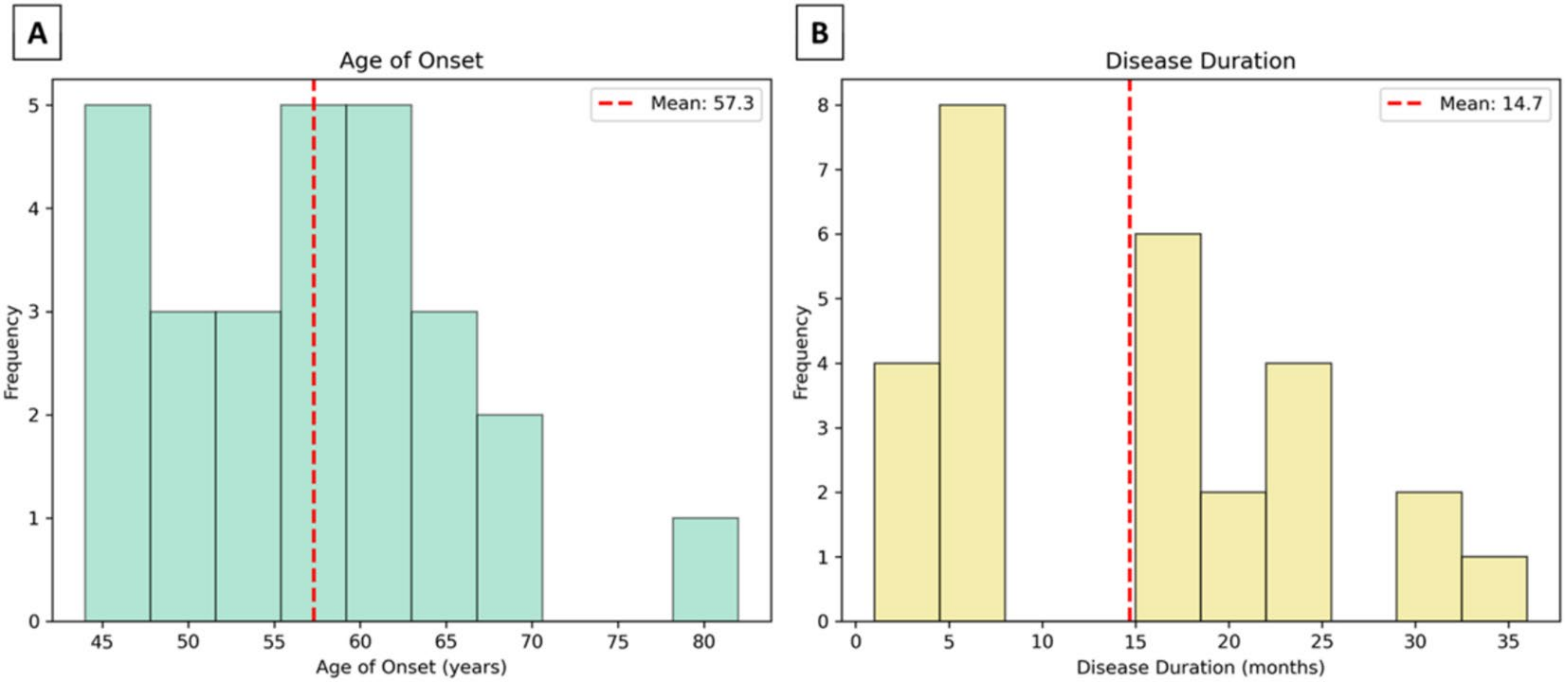
Age of onset and disease duration of FFI in the Portuguese family. **A** The figure shows the distribution of ages at onset for FFI, with frequency represented by the green bars and mean of age onset of 57.3 years (red line). The majority of cases fall within the range of 47–67 years, with some outliers at both younger and older ages (*n* = 27, 43–82 years old). **B** This figure illustrates the distribution of disease duration among affected individuals, with frequency represented by the yellow bars and mean of disease duration of 14.7 months (red line). Most cases had a disease duration between 6 and 18 months, with a few individuals exhibiting prolonged survival up to three years (*n* = 27, 1–36 months)

The age of onset and disease duration were analyzed in 27 out of 34 individuals affected by FFI from the Portuguese family cohort with available data. These cases represent a subset of the Portuguese pedigree tree, as other affected members were excluded due to missing data for one or both of these parameters. The mean age of onset was 57.23 ± 8.89 years (Table 1), ranging from 44 to 82 years old (Fig. 3A). The disease duration varied significantly, ranging from less than six months to three years, with an overall mean of disease duration and SD of 14.58 ± 9.96 (Table 1). In the majority of individuals, it was observed a rapid progression of the disease with a duration ranging from one month to a year. A small number of cases exhibited prolonged disease duration, ranging from more than a year to three years post-onset (Fig. 3B).

**Table 1:** Mean and standard deviation (SD) of age of onset and disease duration of the affected individuals

	Mean age onset (years) ± SD	Mean diseases duration (months) ± SD^a^
Male	57.67 ± 10.70	13.47 ± 8.57
Female	56.66 ± 5.82	16.09 ± 11.87
Total	57.23 ± 8.89	14.58 ± 9.96

^a^Mean diseases duration were determined for patients with unknown absolute number as < 6 was replaced by 3, > 18 was replaced by 16 and > 24 was replaced by 30

### Correlation of age of onset and disease duration

**Fig. 4:**
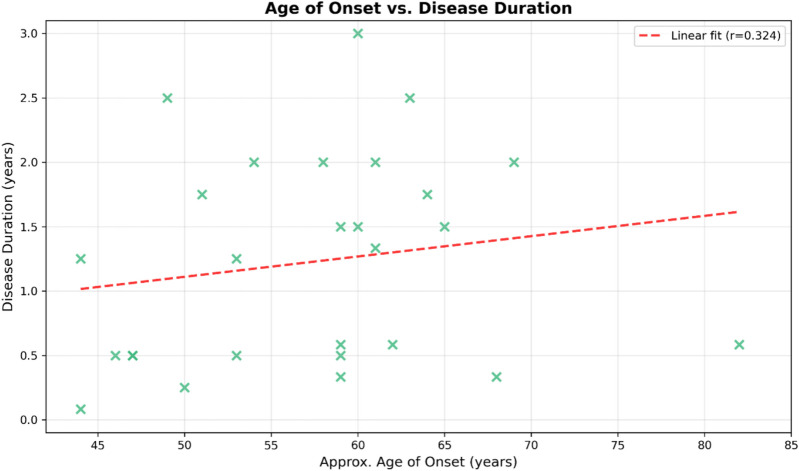
Scatter plot showing the correlation between approximate age of onset (*x* axis) and disease duration in years (*y* axis) for 27 family members with complete data. Each point (×) represents an individual case. The red dashed line shows the linear regression fit (Spearman’s *r* = 0.324, *p* = 0.09)

To further illustrate the relationship between age of onset and disease duration, a scatter plot was generated (Fig. 4). The distribution of data points shows a broad range of disease durations across different ages of onset. The data points do not indicate a correlation between these variables, as the duration of the disease does not appear to be affected by the age at which symptoms first appear.

### Age of onset and disease duration in female and male groups

**Fig. 5:**
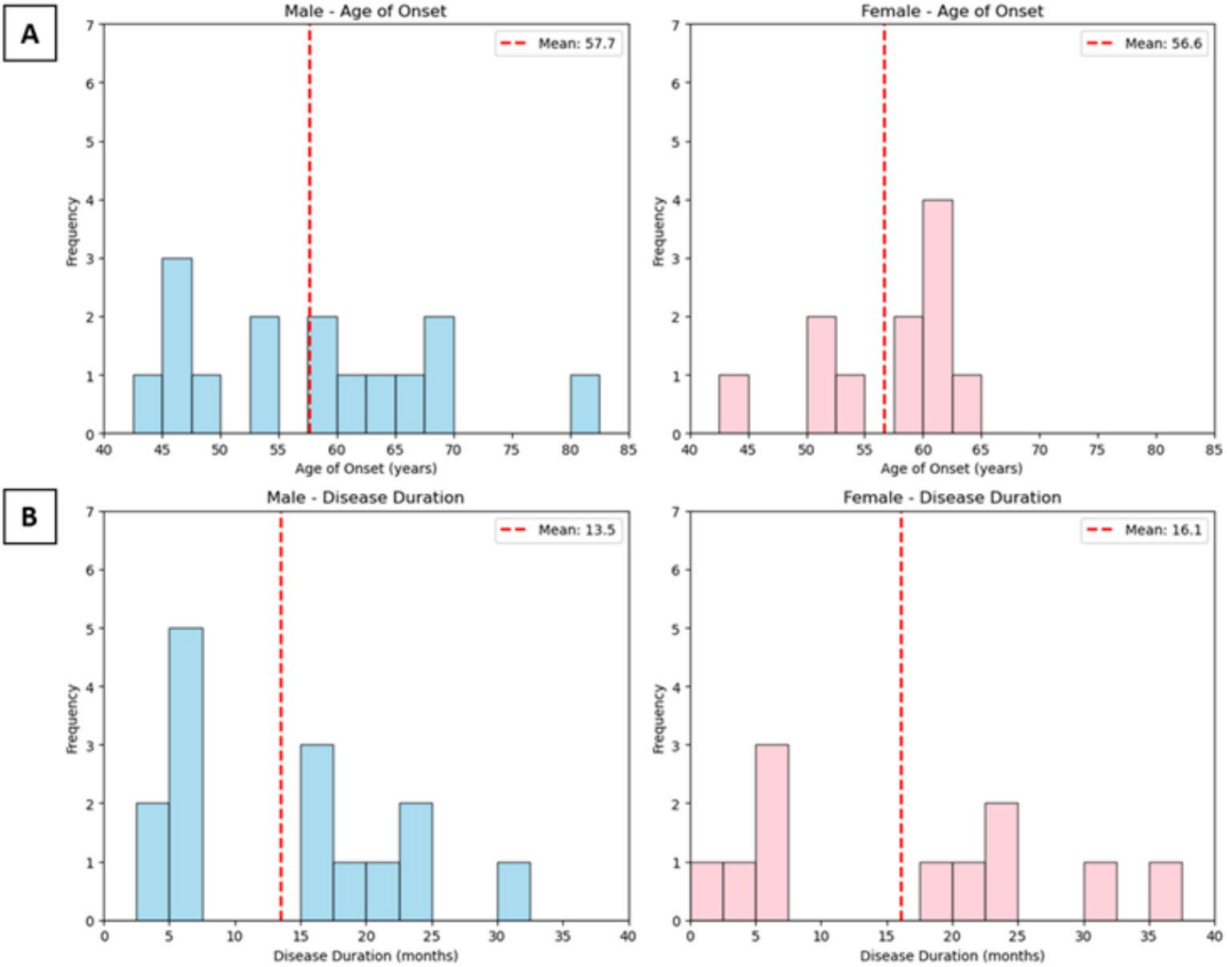
Influence of different gender in the age of onset and disease duration of FFI. **A** Female (*n* = 12) and male (*n* = 15) group showed no significant difference in the age of onset. **B** Female (*n* = 12) and male (*n* = 15) group showed no significant difference in the age of onset

In total, the age of onset and diseases duration from 12 female and 15 males were analyzed. The mean age onset was 57.7 in males and 56.8 in females and the mean of disease duration was 13.5 in males and 16.2 in females. The statistics showed no significant difference in the age of onset between females and males in the age onset and in the disease duration (Fig. 5).

### Prodromal symptoms and clinical manifestations based on family observations

Retrospective clinical and caregiver reports for 9 deceased individuals with genetic confirmed^1^ FFI suggest the presence of symptoms and signs that may represent a prodromal phase of the disease (Table 3). On average, 5 years before the onset of full-blown symptoms, affected individuals begin to experience generalized pain, particularly in the cervical and lumbar regions, accompanied by persistent headaches (9/9, 100%). Behavioral changes, including mood swings and irritability, were universally reported (9/9, 100%). Endocrinological disturbances, including elevated cortisol levels and altered thyroid function (increased levels of anti-thyroperoxidase (TPO) and anti-thyroglobulin antibodies) were documented in 4 out of 7 cases for whom endocrine medical data were available. In 3 cases, family members reported symptom onset coinciding with perimenopause and postmenopausal period in affected women. However, it remains unclear whether this association exists in other affected women due to limited data. Other early reported symptoms included tinnitus (9/9, 100%), pruritus in the dorsal region (7/9, 77.8%), and only one case reported anosmia (1/9, 11.1%).

Approximately two years before diagnosis, all affected individuals exhibited depression, worsening memory and attention deficits, and increasing pain in the cervical and lumbar regions, along with persistent headaches (9/9, 100%). Visual disturbances (e.g., deteriorating visual acuity, diplopia, and strabismus) emerged universally (9/9, 100%), alongside fatigue (9/9, 100%), muscle weakness (9/9, 100%), unexplained fever (9/9, 100%), and over half of the individuals exhibited constipation (5/9, 55.6%). In all affected individuals the cough reflex was suppressed, and yawning is absent (9/9, 100%). Tremors in the head and upper extremities developed in most of the individuals (8/9, 88.9%).

By one year prior to disease diagnosis, all individuals developed more severe symptoms, including dysarthria, hyperhidrosis, gait disturbances, and daily sleepiness develops as a consequence of impairment of night sleeping pattern (9/9, 100%). During brief sleep episodes, involuntary limb movements and vivid dreaming occurred (9/9, 100%). Autonomic dysfunction becomes more pronounced, with all the affected individuals exhibiting symptoms such as lightheadedness, hypertension, and tachycardia (9/9, 100%). Gastrointestinal symptoms, particularly, severe constipation was reported in over half of the affected individuals (5/9, 55.6%). In addition, the majority developed diabetes mellitus (6/9, 66.66%).

At the time of diagnosis, most of the individuals experienced involuntary movements in the lower extremities, myoclonus and a marked worsening of gait disorders, leading to significant balance and coordination issues (8/9, 88.9%). Cognitive decline accelerates, with increasing confusion and restlessness (8/9, 88.9%), accompanied by urinary incontinence or retention (8/9, 88.9%), severe dysphagia, and weight loss (8/9, 88.9%).

In terminal stages of the disease, mortality predominantly resulted from respiratory failure or systemic infections in most of affected individuals (7/9 cases, 77.8%). One patient progressed to akinetic mutism (1/9, 11.1%), while another case experienced sudden death before reaching the terminal stage of the disease (1/9, 11.1%).

All 9 individuals included in this retrospective analysis were confirmed to carry D178N mutation through molecular genetic testing. However, only 4 of these individuals appear in the pedigree shown in Fig. 1, as remaining 5 belong to generation VI, which was excluded from the figure for confidentiality. Of the 9 mutation-positive individuals, only 2 were aware of their genetic status prior to symptoms onset. One female tested more than 10 years before onset, and one male tested a few months before the full clinical manifestation. The remaining 7 individuals were unaware of their mutation status at the time of clinical presentation (Table 2).

**Table 2:**
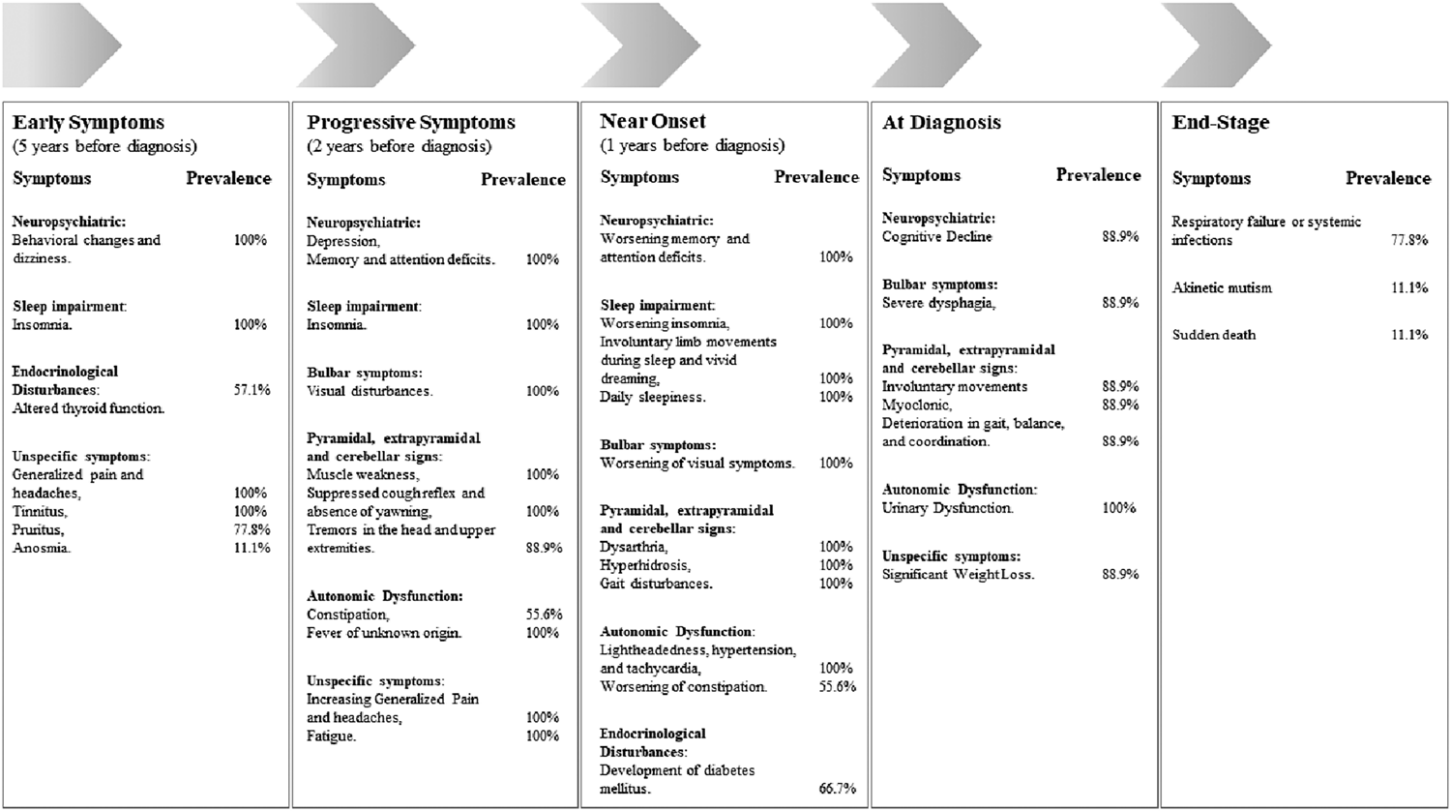
Prodromal symptoms and clinical manifestations based on family observations

## Discussion

### Inheritance patterns and age of onset

The pedigree analysis of a five generation of a Portuguese family affected by FFI, provides essential insights into the inheritance patterns, age of onset, and clinical progression of affected individuals. As with prior reports [29], the data confirm an autosomal dominant inheritance, demonstrated by vertical transmission and multiple affected individuals across generations.

Although genetic anticipation, where age of onset decreases in successive generations, has been proposed in genetic prion diseases [30–32], our findings do not support its consistent presence (Table 3). In fact, we observed a statistically significant increase in age of onset from the third to the fifth generation. Only two isolated branches showed signs or earlier onset, suggesting anticipation is not a reliable feature in this pedigree.

**Table 3:** Mean and standard deviation (SD) of age of onset and disease duration of the affected individuals

Countries	Sample size (*n* =)	Sex (F/M)	Mean age of onset (years)	Mean disease duration (months)	References
Portugal	34	16/18	57.23 ± 8.89	14.58 ± 9.96	Present study
Spain	12	6/6	53 ± 10.03	12.17 ± 4.37	[42]
German	41	13/28	56 (23–73)	11 (6–24)	[43]
Italian	15	N/A	49 (35–61)	13 (7–25)	[29]
Austrian	5	3/2	N/A (20–60)	N/A (8–20)	[32]
China	131	57/72	47.51 ± 12.53 (17–76)	13.20 ± 9.04 (2–48)	[44]

These findings may reflect the underlying molecular basis of FFI. Unlike disorders with repeated expansions (e.g., myotonic dystrophy), FFI results from a point mutation (D178N in the PRNP gene), and lacks the known molecular mechanism of anticipation [33]. Moreover, prior reports (Supplement Table 1) suggesting anticipation [30, 31] may suffer from ascertainment bias [34], where early diagnosis mimics anticipation without reflecting true genetic effects.

### Age of onset and disease duration

In this pedigree, age of onset clustered in the late 50’s, with a mean approximately 57 years, consistent with other well characterized FFI families (Table 3) such as the Italian and Spanish pedigrees [13, 35]. The majority of affected individuals (67%) experienced rapid disease progression, resulting in death within months to 1.5 years. However, a significant subset (33%) exhibited prolonged disease duration, surviving well beyond the typical clinical course reported in the literature, where fewer than 20% of cases reach two to three years [36].

This variability in the age of onset (43–82 years old) and disease duration (1–36 months), suggests that other modifying factors may play a role. Environmental exposures, epigenetic modifications, and stochastic biological processes [37–40] may all contribute to these interindividual differences. In addition, the lack of a correlation between age of onset and disease duration and gender supports the hypothesis that such factors may contribute to the variability of the disease progression.

Interestingly, we identify one individual who developed symptoms only at the age of 82, significantly later than the average onset in this pedigree (~ 57 years). While this does not meet the strict definition of “resilient” [41], which typically refers to asymptomatic carriers reaching advanced age without disease, it may represent a case of partial resilience. No cases of “superresilients” were observed, which means healthy mutation carriers reaching advanced age without symptoms, despite having offspring affected by the diseases.

### Prodromal symptoms, clinical manifestation and potential triggers

The characterization of prodromal symptoms in FFI remains a complex and widely underexplored area, particularly due to the rarity of the disease and the fact that most available prodromal symptoms data derived from retrospective caregiver reports, while subjective, offer valuable insight into the early manifestations of the disease. In this study, caregiver reports suggest a constellation of early symptoms up to five years before the formal diagnosis of the disease, including headaches, cervical and lumbar pain, pruritus, tinnitus, dizziness, and fatigue. These symptoms are often overlooked or misattributed to stress or aging, which may contribute to delays in clinical recognition.

Although unspecific, these early symptoms typically intensified over time and were only reported in individuals who later developed FFI, not in healthy relatives or those who died of unrelated causes. Importantly, most of the individuals described (7 out of 9) were unaware of their genetic status, making it unlikely that their symptom reporting was influenced by expectation or diagnostic anxiety.

Given that FFI diagnosis is based primarily on clinical criteria, the timing of diagnosis is often arbitrary and dependent when symptoms become disruptive enough to prompt evaluation. It is plausible that, in many cases, the actual onset of FFI in this cohort occurred earlier than what was clinically documented. Many individuals delayed seeking medical help in the initial phases, often due to denial, minimization of symptoms, or reluctance to attend hospital appointments. Insomnia, in particular, was reported as one of the earliest symptoms (up to 5 years prior diagnosis) and was noted to worsen over time. This observation poses a conceptual question: where should we draw the line between the prodromal phase and the actual clinical onset?

In the literature, progressive insomnia is widely recognized as hallmark of FFI onset [45, 46]. If we follow this convention, the appearance of insomnia should mark the biggening of the symptomatic phase. However, in this pedigree, insomnia emerged years before a formal diagnosis was made. If these early symptoms are accepted as part of the clinical phase, the disease duration in these individuals would extend far beyond the typical 7–25 months [47], reaching up to 5–8 years. Although this exceeds the most reported range, it’s worth noting that some authors have documented diseases durations as long as 72 months [45]. Importantly, the time of the clinical diagnosis to death in this family remains consistent with typical FFI course [46], confirming that rapid progression occurs once clinical onset is established.

This discrepancy raises an important interpretive question. In our analyses, we have conservatively referred to these early symptoms, including insomnia, as prodromal, primarily because of the length of time between the symptom’s onset and formal diagnosis. This classification is not intended to downplay the potential that these symptoms may actually represent the true onset of disease, but rather to reflect the uncertainty that exists in the early, often ambiguous, stages of illness.

Importantly, the FFI symptoms at onset may vary between individuals, and not all patients necessarily present first with insomnia [47–49]. This heterogeneity complicates efforts to define a single, uniform starting point for the disease and further supports the need for nuanced interpretation of early symptoms.

The clinical manifestation reported by the family members align with the known phenotype of FFI [50], that includes severe progressive untreatable insomnia, autonomic dysfunction, cognitive impairment, and motor disturbances. Interestingly, according to reports from family members, some individuals developed symptoms of the disease following episodes of significant emotional stress or mourning. This anecdotal observation aligns closely with documented cases of diagnosed depression and prior treatment with antidepressant medication among those affected before disease diagnosis [51, 52], suggesting a potential role of psychological stressors in triggering or accelerating disease progression. This correlation is further supported by studies that identify depressive symptoms as prodromal symptoms in prion diseases [53–55].

Lastly, we acknowledge that our data are retrospective and based on family member reports, which introduces potential for bias including recall bias and other confounders that cannot be excluded. However, these prodromal symptoms were not reported for family members who died of other causes, suggesting they may not reflect purely nonspecific recall. Although we cannot definitively determine whether these represent true prodromal manifestations or unrelated comorbidities, we consider this information important for guiding future prospective studies.

### Hormonal changes

Consistent with the previous reports [45], we observed changes in the blood analysis, including elevated morning cortisol levels. As seen in other studies, this patient cohort also exhibited a correlation between disease onset and both amenorrhea and early onset menopause [13] as well as onset of diabetes mellitus [56]. In addition, we detected increased levels of anti-thyroperoxidase and anti-thyroglobulin, autoantibodies commonly associated with autoimmune thyroid diseases [57], up to five years before the disease diagnosis. Although the implications remain unclear, these markers could potentially serve as early indicators of systemic changes preceding the disease onset.

### Global pedigree dispersion

Members of this family pedigree have also migrated to Brazil, France, Switzerland, and Luxembourg. Future studies may benefit from investigating possible genetic links between this linage and other FFI cases worldwide, potentially shedding light on the geographic spread and genetic diversity of the disease.

## Conclusion

This study provides a comprehensive analysis of the inheritance, onset, and clinical progression of FFI within a Portuguese family, reinforcing its high penetrance of the PRNP mutation across multiple generations, with affected individuals present in each generation consistent with previous studies such as those described in Italian, Spanish, and Chinese families. The phenotype observed in this cohort also align with findings from those well-characterized pedigrees, where affected individuals typically develop symptoms in mid-to-late adulthood, followed by a rapid progression of the diseases leading to death within months to a few years.

Notably, the observed variability in disease duration, with a higher proportion of individuals exhibiting prolonged survival as compared to the previous reports, may suggest the potential involvement of genetic modifiers or environmental factors influencing disease progression. Further research is warranted to elucidate these mechanisms and their impact on disease heterogeneity.

In addition, the presence of prodromal symptoms as well as the reported association of onset of the diseases with psychological stressors, underscores the need for heightened clinical surveillance in at-risk individuals. These findings contribute to a growing body of evidence on the phenotypic heterogeneity of FFI and underscore the critical importance of early identification for timely clinical management and potential therapeutic intervention.

Future studies integrating genomic sequencing, biomarkers discovery, and longitudinal clinical assessments will be critical to elucidating the molecular and environmental factors contributing to disease variability. Such investigations may provide novel insights into disease pathogenesis and guide the development of targeted therapeutic strategies for this fatal prion disease.

## Supplementary Information

Below is the link to the electronic supplementary material.

Supplementary file1 (DOCX 49 KB)

## Data Availability

Data supporting the findings of this study are available from the corresponding author upon reasonable request.

## Footnotes

1 Genetic confirmation was obtained for all 9 affected individuals through molecular testing of the PRNP gene. Direct genetic reports were available for 4 cases, confirming the pathogenic variant c.532G>A (p.Asp178Asn) in heterozygosity with M129M in 3 cases and M129V in 1 case. For the remaining 5 cases, D178N mutation status was confirmed through family-reported clinical genetic testing results. Additional genetic analysis in first-degree relatives who are healthy mutation carriers confirmed D178N in cis with M129 configuration and no other rare variants were detected (data not included), supporting the presence of the classic FFI-associated haplotype.
