# Supplementary material for: Pedigree analysis and genetic inheritance of fatal familial insomnia (FFI) in a Portuguese multigenerational family
Source: J Neurol. 2025 Oct 17;272(10):706. doi: 10.1007/s00415-025-13432-2 (PMC12534306; doi:10.1007/s00415-025-13432-2)
Supplement: Supplementary file 1 — Supplementary file1 (DOCX 49 KB) [file 415_2025_13432_MOESM1_ESM.docx]

**Supplement**

**Supplement Table 1.** Age at death and disease of the Affected Individuals in different family three.

(Adapted from: Harder A et al., "Early age of onset in fatal familial insomnia: Two novel cases and review of the literature", Neurogenetics, 2004.)[60]

| Country | Generation  (n) | Onset  (years) | Disease’s duration  (moths) | Age at death | References |
| --- | --- | --- | --- | --- | --- |
| Portugal | III (n=7) | 44 | 1 | 48.00±3.21 | *Present Study |
|  | IV (n=13) | 57.0±9.68 | 8.55±6.32 | 57.31±9.41 |  |
|  | V (n=17) | 55.20±7.47 | 20.13±7.83 | 59.60±7.25 |  |
| Australia | VI (n=6) | 49.83±7.45 | 14.33±5.98 | *^1^50.90±7.08 | [61, 62] |
| Austria | II (n=2) | - | - | 49.00±8.49 | [60] |
|  | III (n=6) | 60±2.83 | 13±7.07 | 53.6±8.2 |  |
|  | IV (n=3) | 27±8.19 | 14.67±4.72 | 27.67±7.64 |  |
| France | I (n=1) | 50 | 6 | *^1^51 | [63] |
|  | II (n=1) | 40 | - | - |  |
|  | III (n=4) | 48±3.56 | 9.67±1.53 | *^1^50±3.61 |  |
| Germany | V (n=1) | 47.5 | 41 | 50 | [64] |
|  | IX (n=5) | 56.7±10.03 | 19.6±17.31 | *^1^58.2±9.15 |  |
|  | X (n=2) | 64.5±8.49 | 6±0 | *^1^65±8.49 | [60] |
|  | XI (n=2) | 44.5±3.54 | 16±11.31 | *^1^46±2.83 |  |
|  | XII (n=4) | 34.75±8.54 | 10.25±2.36 | *^1^35.75±8.54 |  |
|  | XIII (n=1) | 24 | 12 | 25 |  |
| Italy | IV (n=6) | 50.8±6.18 | 11.0±5.48 | 51.8±6.58 | [65] |
|  | V (n=1) | 35 | 25 | *^1^37 |  |
|  | IV (n=1) | 52 | 9 | 53 | [66, 67] |
|  | V (n=4) | 55.75±6.78 | 22.00±5.85 | 58.00±6.10 |  |
|  | I (n=1) |  |  | 58 | [68] |
|  | II (n=1) |  |  | 42 |  |
|  | III (n=1) | 51 | 14 | *^1^52 |  |
| China | I (n=1) | - | - | 49 | [69] |
|  | II (n=1) | - | - | 79 |  |
|  | III (n=3) | - | 12±0 | 45.33±6.66 |  |
|  | IV (n=1) | - | - | 37 |  |
| Korea | I (n=1) | - | - | 40 | [70] |
|  | II (n=2) | - | - | 46±8.49 |  |
|  | III (n=2) | - | - | 51±22.63 |  |
|  | IV (n=3) | - | - | 36.5±2.12 |  |
| Spain | II-4 | 47 | 5 | *^1^47 | [57] |
|  | III (n=2) | 44±5.66 | 8.5±2.12 | 45±5.66 |  |
| USA | III (n=3) | 64.33±6.66 | 9.67±4.04 | *^1^65.33±6.66 | [71] |
|  | IV (n=3) | 44±2.65 | 11.33±6.03 | *^1^44.67±2.52 |  |
|  | V (n=1) | 25 | 12 | *^1^26 |  |

*^1^ The age at death was estimated by adding age onset to the duration of the diseases.

**Supplement table 3.** Age of onset, disease duration, and age at death in affected individuals from the FFI pedigree.

| Pedigree  number | Sex | Approx. age  of  onset (years) | Disease  duration (moths) | Age at  death  (years) |
| --- | --- | --- | --- | --- |
| III-2 | M | - | - | 48 |
| III-4 | M | - | - | 52 |
| III-6 | M | - | - | 48 |
| III-9 | F | - | - | 50 |
| III-11 | F | 44 | 1 | 45 |
| III-13 | F | - | - | 43 |
| III-18 | F | - | - | 50 |
| IV-1 | M | 47 | 6 | 48 |
| IV-3 | M | 46 | 6 | 47 |
| IV-11 | M | 47 | 6 | 48 |
| IV-12 | F | 60 | 18 | 62 |
| IV-17 | F | 59 | 7 | 60 |
| IV-24 | F | 50 | 3 | 50 |
| IV-26 | M | 58 | 24 | 59 |
| IV-28 | F | 62 | 7 | 62 |
| IV-29 | M | 82 | 7 | 82 |
| IV-34 | F | - | - | 46 |
| IV-36 | F | 59 | 6 | 60 |
| IV-38 | M | 59 | 4 | 59 |
| V-2 | F | 61 | 24 | 62 |
| V-3 | M | 69 | 24 | 70 |
| V-6 | F | 63 | 30 | 65 |
| V-7 | M | 49 | 30 | 50 |
| V-21 | M | 65 | 18 | 67 |
| V-31 | F | 54 | 24 | 55 |
| V-33 | F | 60 | 36 | 63 |
| V-38 | M | 53 | 6 | 54 |
| V-41 | F | 59 | 18 | 61 |
| V-42 | M | 64 | 21 | 65 |
| V-44 | M | 61 | 16 | 63 |
| V-50 | M | 53 | 15 | 54 |
| V-52 | M | 44 | 15 | 45 |
| V-54 | M | 68 | 4 | 68 |
| V-68 | F | 51 | 21 | 52 |
